# Rapid mixing of viscous liquids by electrical coiling

**DOI:** 10.1038/srep19606

**Published:** 2016-02-10

**Authors:** Tiantian Kong, Jingmei Li, Zhou Liu, Zhuolong Zhou, Peter Hon Yu Ng, Liqiu Wang, Ho Cheung Shum

**Affiliations:** 1 Guangdong Key Laboratory for Biomedical Measurements and Ultrasound Imaging, Department of Biomedical Engineering, Shenzhen University, 3688 Nanhai Avenue, Shenzhen 518060, China; 2 Department of Mechanical Engineering, the University of Hong Kong, Pokfulam Road, Hong Kong; 3 HKU-Zhejiang Institute of Research and Innovation (HKU-ZIRI), Hangzhou, Zhejiang 311100, China; 4 HKU-Shenzhen Institute of Research and Innovation (HKU-SIRI), Shenzhen, Guangdong; 5 ASM Pacific Technology Ltd, 16 Kung Yip Street, Kwai Chung, Hong Kong

## Abstract

The control for the processing of precursor liquids determines whether the properties and functions of the final material product can be engineered. An inherent challenge of processing viscous liquids arises from their large resistance to deform. Here, we report on the discovery of an electric approach that can significantly contribute to address this challenge. The applied electric force can induce a straight viscous jet to coil, and the resulting coiling characteristics are governed by the electric stress. We demonstrate the promising use of electrical coiling in the rapid and efficient mixing of viscous liquids. Remarkably, the degree of mixing can be precisely adjusted by tuning the applied electric stress. Our approach of controlling the coiling electrically has important implications on applications such as dispensing and printing of resins, printing patterned surfaces and scaffolds, processing of food and generating non-woven fabrics.

A viscous liquid jet coils and forms a beautiful helical structure resembling a pile of ropes, when dispensed at a distance onto a substrate. This intriguing phenomenon, sometimes known as “liquid rope coiling”, has been extensively studied^1^,^2^,^3^,^4^,^5^,^6^. The driving force of coiling is an axial compressive stress that bends the viscous liquid jet^1^,^2^,^3^,^4^,^5^,^6^,^7^,^8^. This compressive stress arises from the deceleration of the jet along the axial direction^7^,^9^, for example, when a falling liquid jet encounters a solid substrate.

Subjected to a sufficiently large compressive stress, a viscous liquid jet is bound to coil. The coiling of viscous liquid jet is usually a steady-state motion and can be predicted^3^,^4^,^6^,^10^. A coiling liquid jet comprises two parts: a vertical tail, and a helical coil formed as a result of jet bending^1^,^2^,^3^,^4^. The coiling frequency, Ω, is governed by the kinematic viscosity, injection speed and geometric features of the liquid jet, such as its diameter and length^1^,^2^,^3^,^4^. The jet length, which equals the dispensing height, *h,* from the nozzle to the substrate, is the most effective parameter that controls the coiling frequency^2^,^4^,^6^,^8^. After the liquid jet exits the nozzle, it accelerates vertically due to gravity. The jet becomes thinner in radius, *r*, as it travels at a higher velocity, 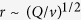, where *Q* is the volumetric flow rate of the jet phase. As the thinning jet approaches the substrate and coils periodically, the jet velocity *v* can be obtained by multiplying the coiling frequency, Ω, by the amplitude, *R*: 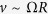. Therefore, the coiling frequency, Ω, strongly depends on the jet radius, which changes sensitively with the dispensing height, *h*: 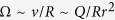. At small *h*, the jet velocity is also small and the jet radius is similar to the nozzle radius^2^,^4^,^6^. A small velocity suggests a small coiling frequency. At large *h*, the jet attains a higher velocity and thus a smaller radius upon contact with the solid substrate. The decrease in jet radius facilitates an increase in the coiling frequency, since the coiling amplitude does not usually increase as the jet radius decreases^1^,^2^,^4^. As such, the coiling frequency can be manipulated by tuning the jet velocity, via varying the dispensing height, *h*.

The control of coiling is crucial for manipulating viscous liquids in printing and dispensing applications^11^,^12^,^13^,^14^. For example, the well-defined attributes of coiling can be utilized to print tunable helical structures^15^,^16^,^17^,^18^,^19^,^20^,^21^,^22^,^23^. Moreover, the coiling can be used to dispense precursor liquids to form spring-like antenna for micro-electronics^11^,^13^, pre-mixed epoxy resins to bond electronic components onto integrated electric circuit^14^, and precursor solutions of hydrogel to fabricate scaffolds and arrays^12^. However, tuning of coiling by varying dispensing height requires the reconstruction of the whole dispensing setup, and an inconveniently long distance between the dispensing nozzle and the substrate. For instance, for a jet with a viscosity of 10^3^ centistoke, a dispensing height of around 140 millimeter is needed for steady coiling^3^. Thus, coiling-based printing becomes impractical for many applications where the dispensing height must be limited to the micro-meter range^15^,^16^,^17^,^18^,^19^,^20^,^21^,^22^,^23^,^24^,^25^.

Electric stress can change both the shape and dynamics of liquid jets significantly. For example, in electrospray for fabricating nano-sized particles, the electrostatic stress results in a fine jet, which subsequently breaks up into nanometer-sized drops^26^. In electrospinning, a tensile electric stress stretches a liquid jet to form nanometer-sized fibers^5^,^27^. Despite its adoption in these established fabrication techniques, to the best of our knowledge, electrical stress has not been investigated as a means to control the coiling of viscous liquid jets.

In this work, we demonstrate that the coiling of viscous liquid jets is highly sensitive to an applied electric stress, which induces coiling at a significantly reduced dispensing height. Under an electric field, the charged viscous liquid jet is stretched and starts to coil at a frequency that increases with an increasing electric stress. The electrical coiling is exploited to achieve rapid mixing between highly viscous liquids (>3 *Pa.s*). We further show that the mixing quality of the viscous liquids can be controlled by varying the applied electric stress. Our electrical coiling approach can inspire new ways to dispense and mix viscous liquids for printing plastic molds and patterned surfaces, as well as for bonding components onto integrated circuit boards.

## Results

### Behaviors of a charged viscous jet in an electric field

**Figure 1:**
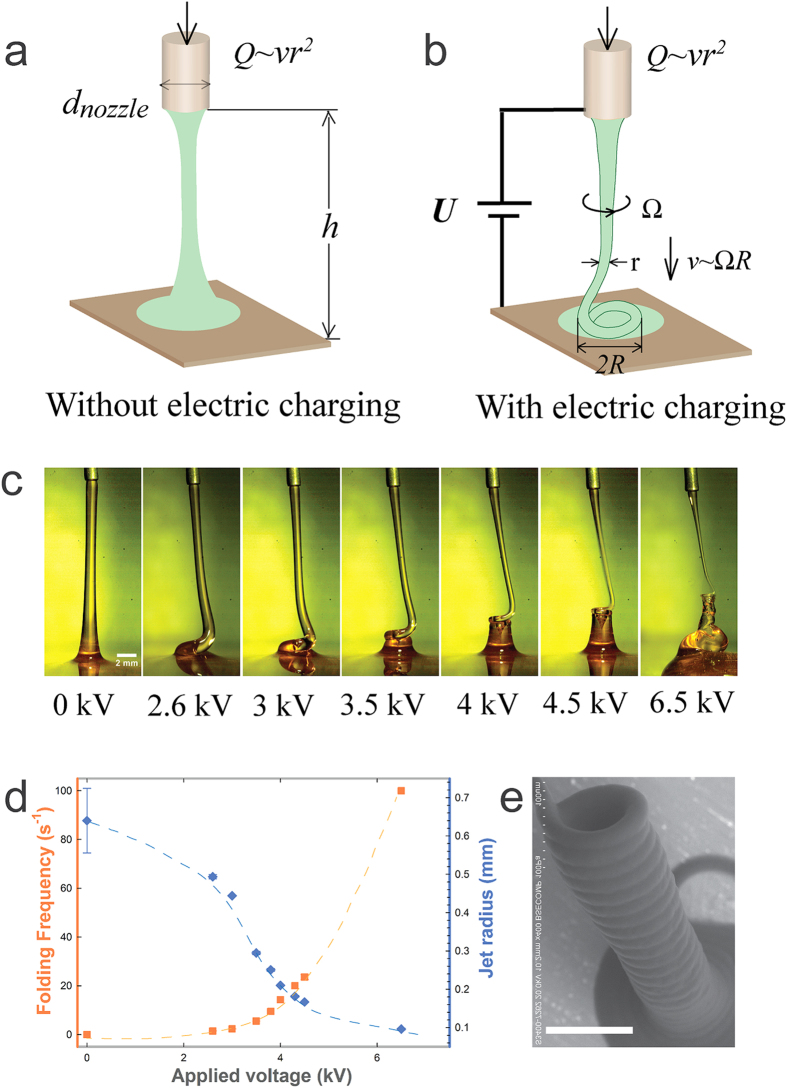
Viscous liquid stream (**a**) forms a straight jet without electric charging (**b**) is induced to coil under an applied voltage; (**c**) A series of high speed camera images showing the coiling of a viscous jet, lecithin from soy bean, under different applied voltages from 0 *kV* to 6.5 *kV* and the same dispensing height, 18 *mm*; The injection flow rate is 50 *ml/h* and the scale bar is 2 *mm*; (**d**) A plot of the coiling frequency and jet radius as a function of the applied voltage, where the trend is represented by the dash lines; (**e**) Scanning electron microscopy (SEM) image of a solidified pillar formed from the coiling of a viscous jet at an applied voltage of 3 *kV* and a dispensing height, 10 *mm*. The dispensed viscous jet in this image is 18 *wt%* polycaprolactone (PCL) in chloroform. Chloroform is quickly evaporated as the jet approaches the grounded plate, leaving behind a solidified polycaprolactone pillar. The scale bar is 500 *μm*.

The applied electric stress at the axial direction can set a straight viscous jet to coil, as shown schematically in Fig. 1a,b (See also Fig. S1 in Supplementary information). To demonstrate the effect of electric stress on the jet morphology, we first form a straight viscous liquid jet without applying any voltage (Fig. 1c, 0 *kV*). Afterwards, we gradually increase the applied voltage and monitor the corresponding morphology of the jet. Surprisingly, we find that, as the applied voltage, *U*, is increased to a threshold value, the viscous jet starts to coil slowly (Fig. 1c, 2.6 *kV*). A sufficiently slender jet can coil under a sufficient compression^28^,^29^. Here, by applying a voltage, the liquid jet is charged and subjected to an electric force that is in the same direction of the flow. The electric force, overcoming the viscous and surface tension forces^26^, accelerates the jet and induces two effects: The jet becomes more slender; and the compression increases as the jet contacts the substrate with a higher terminal velocity (Fig. 1c,d). Both effects facilitate the coiling of the jet. Therefore, at a threshold value of the applied voltage, a straight viscous jet will be set to coil as it becomes sufficiently slender and is subjected to a sufficient compression due to the impact with the substrate. Such coiling differs from whipping in both the visual appearance and the underlying mechanism. Whipping, where a jet chaotically spins, occurs due to the charge repulsion; while coiling, where a jet rotates steadily to form helical structures, arises from the compression of a slender jet^27^,^30^.

For a coiling viscous jet, increasing electric stress leads to further jet thinning and faster coiling. Under a constant flow rate, a decrease in jet radius can result in an increase in the coiling frequency, 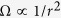. When the electric stress increases, the jet is further accelerated and thinned to achieve a smaller radius *r*, thus leading to a faster coiling. Indeed, as the applied voltage increases from 2.6 *kV* to 6.5 *kV*, the jet radius decreases from 0.5 *mm* to 0.1 *mm*, and the corresponding coiling frequency increases from 2.3 *s*^−1^ to 100 *s*^−1^ , as shown in Fig. 1c,d (See also Fig. S2 of section II in Supplementary information). With a large applied electric force, the viscous jet coils so fast that it piles up to form a pillar with a constant radius (See Movie 1 and Fig. 1e).

Normally, when the length of a jet exceeds its circumference, it tends to break up due to the Plateau–Rayleigh instability^31^. For example, a lecithin jet with radius around 0.4 *mm* breaks up into droplets at a jet length of ~25 mm (See section III in Supplementary information). Interestingly, when a jet is subjected to an electric field, the axial electric stress can completely suppress the Plateau–Rayleigh instability^32^ and thus prevents jet breakup. An otherwise unstably thin lecithin jet can now adopt a radius of around 0.1 mm under the same conditions, in the presence of an applied electric field (Fig. 1c,d). Therefore, varying the applied electric stress can change the jet radius and coiling frequency conveniently and efficiently, under the same dispensing height *h*.

### Scaling analysis of the coiling characteristics of viscous jet

To understand the effects of the electric field on the liquid coiling quantitatively, we analyze the dominant forces acting on the coiling jet. The electric force per unit jet length, *F*_*e*_, scales as 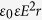
17, where 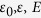 are the permeability of vacuum, the dielectric constant of the liquid and the electric field intensity respectively. Gravitational force, *F*_*g*_, per unit length scales as 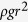, where *g* is the gravitational acceleration. The inertial force, *F*_*I*_, per unit length due to centripetal and Coriolis accelerations scales as 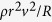, where *ρ* is the density of the liquid jet^1^,^2^. As the liquid jet coils, the viscous force, *F*_*v*_, that resists the deformation of bending and twisting scales as 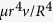
^ ^2,4,6. Based on typical parameters in our experiments (*r* ~ 10^−4^–10^−5 ^*m,* 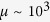 *Pa.s*), we find that while the ratio of gravitational to electric force 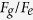 and that of inertial to electric force 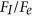 are on the order of 10^−3^ and 10^−2^ respectively, the electric force is comparable with viscous force, 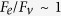. Thus the gravitational and inertial forces are negligible, in comparison to the electrical and viscous forces.

For the liquid jet to rotate and coil steadily with a constant radius *R*, the overall torque must be balanced. The balance between the electric torque and viscous torque yields 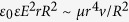. By substituting 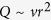
and 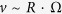, a scaling law for the coiling frequency can be deduced:


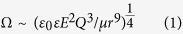


**Figure 2:**
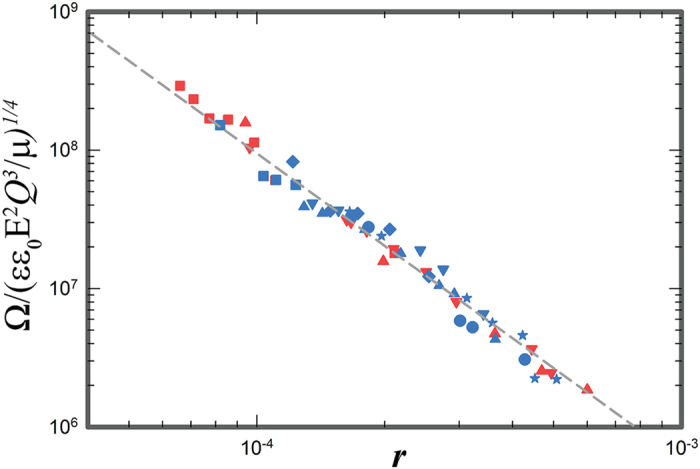
A log-log plot of 
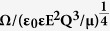
 against jet radius, *r,* based on the scaling law (1). The red and blue symbols correspond to different viscous liquids, lecithin from soy bean and polyglycerolpolyricinoleate (PGPR), respectively. The shape of symbols corresponds to different flow rates of 10 *ml/h* (◼), 20 *ml/h* (◆), 30 *ml/h* (▼), 50 *ml/h* (▲), 70 *ml/h* (●), 150 *ml/h* (★), respectively. The size of the nozzle used for flow rates of 10 *ml/h*, 20 *ml/h*, 30 *ml/h* and 50 *ml/h* is 0.92 *mm*, and that for flow rates of 70 *ml/h* and 150 *ml/h* is 1.4 *mm*. All data are fitted by the relation 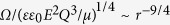 , represented by the grey dash line, confirming the scaling law (1).

To test this prediction, we examined the dependence between the coiling frequency and the jet radius by varying the electric field intensity and volumetric flow rates, using different viscous liquids. All experimental data falls onto a line fitted by a power law 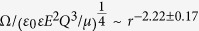, which agrees well with the theoretically predicted scaling law (1) as shown in Fig. 2.

### Formation of ring patterns from the coiling of a compound jet comprising two viscous liquids

**Figure 3:**
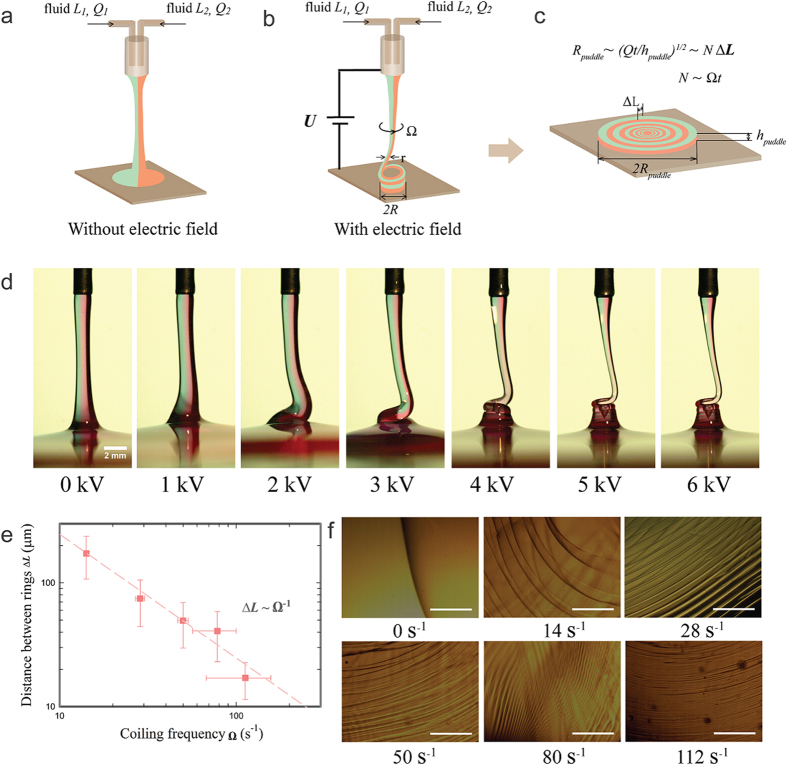
Schematic showing two neighboring touching viscous jets flow in parallel (**a**) forms a straight compound jet in the absence of applied voltage; (**b**) the compound jet is induced to coil with an applied voltage; (**c**) Schematic shows the deposited puddle on the substrate. (**d**) A series of high speed camera images showing the compound jet, comprising two dyed silicone oils, coils under different applied voltage from 0 *kV* to 6 *kV* and the same dispensing height, 13 *mm*. A distinctive interface exists between the green and red regions. The scale bar is 2 *mm*. (**e**) A log-log plot of 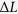
as a function of Ω; the dash line shows a power-law fit to the data with an exponent of -1, confirming our theoretical prediction 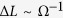. (**f**) Optical microscope images of the ring pattern from the resin coils spreading onto the collection plate under different applied voltages, 0 *kV*, 4 *kV*, 5 *kV*, 6 *kV*, 7 *kV*, 8 *kV*, respectively. The compound viscous liquids used here are two parts of a reactive epoxy resin. The reaction between resins typically takes a few minutes, while the spreading of resins typically takes seconds. Thus, the resins spread to form a flat puddle on the substrate long before solidification is completed. The flow rates of the two parts of an epoxy resin are kept at 15 *ml/h* for all the images. The same amount, 400 *μL*, of the epoxy resins was collected in all samples. The scale bar is 500 *μm*. All the images of the deposited puddle on the collection plate are taken immediately after the jet comes into contact with the substrate.

As a viscous jet coils under an electric field, more liquid surface area is created. If a compound jet comprising of two viscous liquids is set to coil, the increase in surface area between these two liquids as a result of coiling can potentially be harnessed to promote their mixing (Fig. 3 and Movie 2 in Supplementary information). Due to the large viscosities and the consequently low Reynolds number, the flow of the two viscous liquids is laminar^33^. A sharp interface between these two liquids is observed in the absence of coiling (See Fig. S4a,b in Supplementary information). Intriguingly, when the compound jet is set to coil under electric field, the interface between these two liquids forms a ring pattern (See Fig. S4c,d in Supplementary information). Within this ring pattern, the distance, 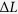, between the neighboring liquid phases is significantly reduced, comparing to the case of no coiling. In this manner, the coiling of compound viscous jets may potentially enhance the mixing between them, which usually takes place extremely slowly for low Reynolds number flows^33^,^34^.

More interestingly, the distance between the rings, 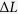, strongly depends on the coiling frequency of the compound viscous jet. As the compound viscous jet coils faster under an increasing applied voltage as shown in Fig. 3d, the ring pattern with a smaller distance 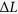 will be formed. The coiled compound liquid jet deposits onto the substrate and spreads to form a puddle with a radius, 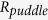, and a height, 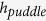, as illustrated in Fig. 3c. Due to mass conservation, the radius of the deposited puddle is 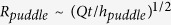. Meanwhile, as the jet coils, the number of rings, *N,* scales linearly with the coiling frequency, Ω, and the time, *t*: 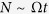. Moreover, the radius of the deposited puddle 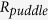 can be estimated as: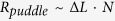, thus giving 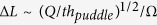
(Fig. 3c). Therefore, 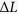 should scale linearly with the reciprocal of the coiling frequency, under constant flow rate, collection time and fixed height of the puddle^35^. To verify this scaling relationship, we compare the ring patterns formed from the coiling of the same compound viscous jet at different frequencies. Indeed, in a log-log plot of 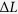 against the coiling frequency, Ω, the data collapse onto a straight line with a slope of −1, confirming our prediction, 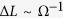, in Fig. 3e,f. At a low coiling frequency, the distance between the rings can be observed macroscopically with naked eyes. However, at a high coiling frequency, the distance between the rings are too small to observe without resorting to high-magnification microscopy (Fig. 3f). These results suggest that the distance between different liquids, represented by 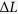, can now be tuned by the coiling frequency of the compound jet. Therefore, the electrical approach can facilitate the use of coiling for mixing by enabling the tuning of the coiling frequency precisely and efficiently.

### Quantifying the mixing between two viscous liquids by electrical coiling

Based on the above results, the distance between different liquids 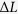 can be precisely tuned through varying the electric voltage applied to coil the viscous liquid jets; we propose exploiting the exquisite control to promote mixing. To confirm and quantify the mixing by the proposed electric coiling approach, we choose two reactive epoxy resins. The two resins react with each other upon contact, producing heat while solidifying and curing in a diffusion-controlled exothermic reaction. The effectiveness in promoting mixing can be demonstrated and quantified by the heat flux generated from the reaction. For diffusion-controlled reactions, the produced heat flux, *q,* during reaction scales linearly with the diffusion flux, *J,* of the reactants, *q* ~ *J*. Moreover, by Fick’s first law, the diffusion flux, *J,* scales linearly with the reciprocal of the distance, 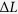 (See Fig. S5 and section V in Supplementary information). Therefore, the scaling relationship between the generated heat flux, *q,* and the distance, 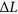, can be expressed as follows: 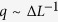.

**Figure 4:**
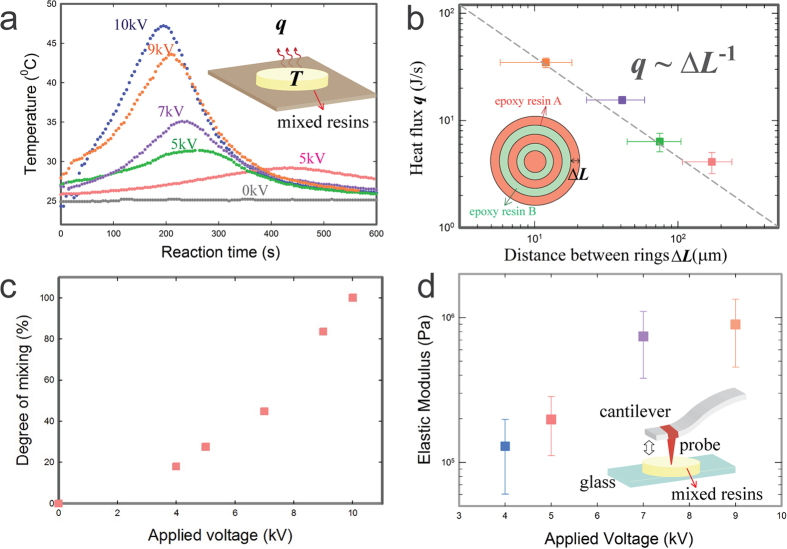
(**a**) A plot of temperature against time under different applied voltages. The rise in temperature, *T*, indicates the initiation of reaction while the drop in temperature is due to heat dissipation to the ambient environment. The inserted schematic shows that the mixed two parts of epoxy resins reacts with each other and thus generates heat. (**b**) A log-log plot of the generated heat flux, *q,* as a function of 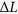. The dash line represents a power-law fit with an exponent of –1, confirming our theoretical prediction 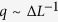. (**c**) A plot of the degree of mixing as a function of applied voltages. (**d**) A plot of the elastic modulus of the mixed epoxy as a function of different applied voltages. The inserted schematic shows the mechanical properties of the resulting mixtures are probed by a modified nano-indentation method.

To confirm the predicted scaling relationship, 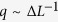, we tune 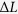 by varying the applied voltage, and monitor the temperature change during the reaction. Without an applied voltage, the compound epoxy resin does not exhibit any coiling. Consequently, mixing between different resins is extremely slow, as reflected by the absence of any temperature change during the time of observation. With an increased applied voltage, the compound jet coils faster, and the temperature rises within a shorter time, as shown in Fig. 4a. Using the internal heat equation and Newton’s law of cooling, the generated heat flux we estimate based on the temperature change is in excellent agreement with the predicted scaling relationship, 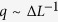, as demonstrated in Fig. 4b (See section VI in Supplementary information). In addition, the degree of mixing is defined as the ratio of the generated heat of incompletely mixed reactive resins to that of the completely mixed resins. The increased applied voltage leads to a higher degree of mixing for the same period of time, as demonstrated in Fig. 4c. (See section VII in Supplementary information).

Moreover, the significant indirect role of the electric stress on the mixing through reducing 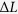 is also manifested in the property of the resultant mixture. Without electrical charging of the epoxy, the poor mixing and incomplete reaction leads to unsolidified epoxy, as exhibited by the inability to adhere a metal rod to a plate (See Fig. S7a in Supplementary information). When the epoxy resins are charged with an applied voltage of 8 *kV*, they are thoroughly mixed and reacted, as indicated by the solidified mixture that attaches the metal rod to the plate (See Fig. S7b in Supplementary information). The mixing effect induced by the electrical charging can also be examined through the mechanical strength of the mixed resultant mixture. Our result demonstrates that an increase in the applied voltage can lead to a corresponding increase in the elastic modulus of the mixed epoxy resins, as shown in Fig. 4d. All these results confirm the effectiveness of electrical coiling in enhancing mixing and reaction of viscous liquids and in controlling the properties of the reaction product, highlighting the potential of this approach for achieving dispensing, mixing and printing structures with tunable modulus in one step.

In conclusion, we introduce an electric approach that can control the coiling of viscous liquids efficiently, rapidly and conveniently. The electric stress can induce and enhance coiling, by dramatically reducing the jet radius and increasing the coiling frequency. We report a simple scaling law that accurately predicts the coiling frequency based on the balance between the electric compressive and viscous resistance torques. Furthermore, we exploit this highly controlled electrical coiling to achieve rapid mixing between different viscous liquids dispensed in parallel from an electrically charged nozzle. The degree of mixing is precisely directed by the applied electric stress, as confirmed by our demonstration using a diffusion-controlled exothermic curing reaction. Our approach is active, efficient and facile with low energy consumption due to the negligible current generated. This approach is expected to make an impact in industries that rely on processing of highly viscous liquids, such as food, polymer and viscous printing industries.

## Methods

### Set-up for dispensing viscous liquids

We fabricated nozzles to inject one or multiple liquids in parallel (Figs 1a,b and 3a,b). The diameter of the nozzle varied from 20 *μm* to about 10 *mm*. Under the same dispensing height, jets with a larger average radius can be formed using a larger nozzle. The nozzle was fabricated by assembling metal tubes or glass capillaries (See Fig. S8 and section IX in Supplementary information). A metal plate or indium tin oxide (ITO)-coated glass slide was placed below the nozzle to collect the liquids. One or multiple viscous liquids were injected through a nozzle of diameter, 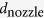, from a height, *h,* using syringe pumps (Longer Pump). The injected liquid phase was charged by connecting the injection metallic nozzle to the positive electrode of a direct current (DC) high voltage power supply, and grounding the collection plate placed below the injection nozzle. The direction of the electric field generated was the same as the flow direction of the viscous jet. The corresponding electric field intensity was controlled by adjusting the potential value of the power supply, *U,* while the distance between electrodes were kept constant. The calculated electric field intensity typically ranged from 0 *kV/cm* to 6 *kV/cm*. The formation of Taylor cone was not observed in this range of the electric field intensity. The electrically induced coiling of viscous liquids was visualized and recorded using a high speed camera (Phantom V 9.1) coupled with a zoom lens (Nikon).

To employ the electrical coiling for mixing, two different viscous liquids, *L*_*1*_ and *L*_*2*_, were dispensed under constant flow rates (Fig. 3a,b). Due to the high viscosity (>3 *Pa.s*) of the liquids and small size (0.02 ~ 2 *mm*) of the device, the corresponding Reynolds number was on the order of 10^−2^ or below, leading to a laminar flow. Therefore, the liquids flowed in parallel with a distinctive interface between each other to form a compound jet in the absence of any applied voltage (Fig. 3d, Fig. S4a,b). As the applied voltage was increased to a threshold value, the compound jet was induced to coil vigorously (Fig. 3d, Fig. S4c,d).

The coiling frequency, Ω, and jet radius, *r,* were obtained after processing and analyzing the high-speed images and videos using an open-source image-processing software, Image J (version: 1.48v). The frame rates of the camera were 200 *frames·s*^−1^, 600 *frames·s*^−1^, 1200 *frames·s*^−1^ for the low, moderate and high coiling frequencies, respectively. We counted the frames during which the liquid jet completed a coil and calculated the corresponding coiling frequency. Each frequency was based on measurements from at least five different coiling cycles while each jet radius was measured using at least five high-speed images.

### Composition of the viscous liquid phases

The liquids employed in all experiments had a viscosity higher than 3 *Pa.s*; these included commercially available epoxy resins (Pattex, 5-minute), polydimethylsiloxane (Dow Corning), polyglycerolpolyricinoleate (PGPR, ILshinwells co., Ltd), silicone oils (Aladdin). To facilitate observation of the interface, two methanol-soluble dyes, Oil red O (Sigma-Aldrich) and Malachite Green (Aladdin), were added to silicone oils for visualization under optical microscopy respectively.

The viscosities of the liquids used in our experiments were measured using a rheometer (Brookfield, R/S series) and were listed and summarized (See Fig. S9 and Table S10 in Supplementary information). All the viscous liquids we used in this manuscript were Newtonian, and thus non-Newtonian effects were not taken in account.

### Demonstration of mixing between viscous liquids

To demonstrate the significant enhancements in the speed and degree of mixing by electric coiling, commercially available two-part epoxy resins (Pattex, 5 minute) were used. These two parts of the epoxy resins reacted with each other and solidified after an exothermic reaction. We monitored the temperature change during the mixing and the ensuing reaction for ten minutes. The change in temperature was controlled by the extent of the reaction. Subsequently, we measured the elastic property of the mixed and solidified epoxy by nano-indentation using an atomic force microscopy that consists of a Zeiss microscope integrated with a JPK NanoWizard II controller (JPK instruments AG, Germany); the modulus was analyzed and calculated based on a rate-jump method described previously^36^,^37^.

## Additional Information

**How to cite this article**: Kong, T. *et al*. Rapid mixing of viscous liquids by electrical coiling. *Sci. Rep.* **6**, 19606; doi: 10.1038/srep19606 (2016).

## Supplementary Material

Supplementary Information

Supplementary Movie 1

Supplementary Movie 2
