# Supplementary material for: Rapid mixing of viscous liquids by electrical coiling
Source: Sci Rep. 2016 Feb 10;6:19606. doi: 10.1038/srep19606 (PMC4748218; doi:10.1038/srep19606)
Supplement: Supplementary Information [file srep19606-s1.doc]

Supplementary Information

Rapid mixing of viscous liquids by electrical coiling

Tiantian Kong, Jingmei Li, Zhou Liu, Zhuolong Zhou, Peter Hon Yu Ng, Liqiu Wang* and Ho Cheung Shum*

*corresponding author: [lqwang@hku.hk](mailto:lqwang@hku.hk), [ashum@hku.hk](mailto:ashum@hku.hk)

1. **Inducing coiling by charging the viscous liquid jet**


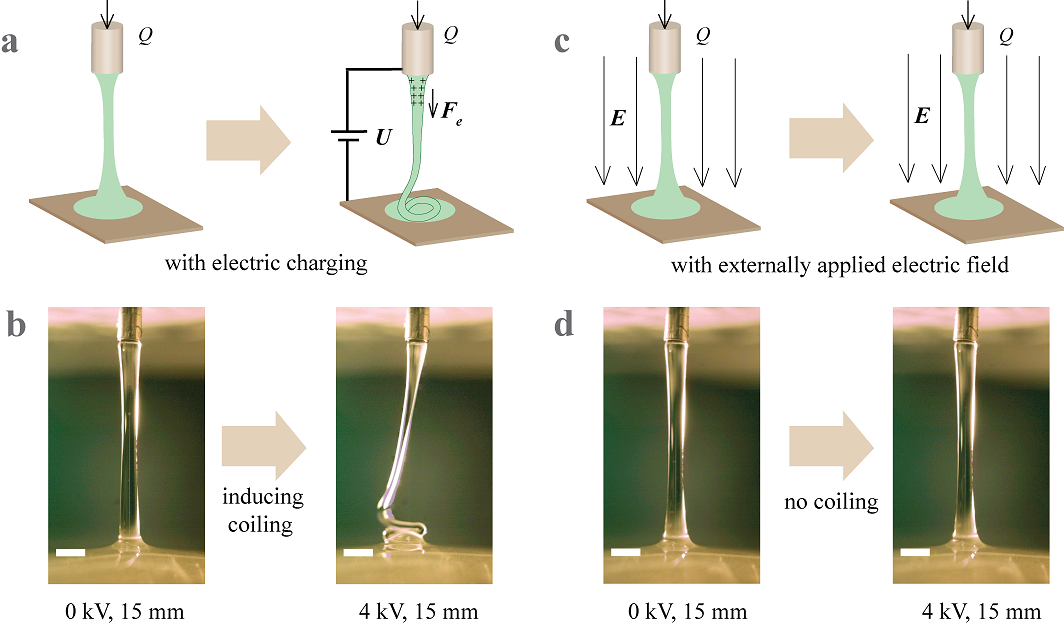


**Figure S1.** (a) Schematic and (b) high speed camera images showing that a straight viscous jet is induced to coiling by charging the liquid with an applied voltage of 4 *kV*; (c) Schematic and (d) high speed camera images showing that a straight viscous jet is not responding to an externally applied electric field without it being directly charged.

1. **The measurement of current generated during electrical coiling**

**
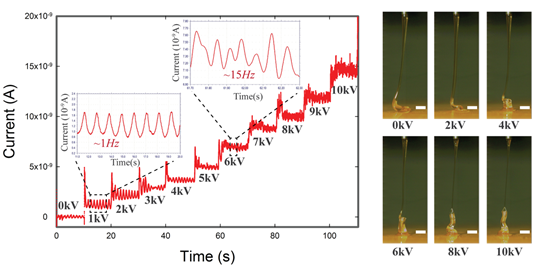
**

**Figure S2.** The measured current (left) and high speed camera images (right) of a coiling lecithin jet under different applied voltages by an electro-chemical work station (CH Instrument, Model 660E). The applied voltage increases by 1 *kV* every 10 seconds, while the coiling frequency ranges from 1*s^-1^* (1 *kV*) to 120 *s^-1^*(10 *kV*). The dispensing height is kept at 21*mm*. The fluctuations in the current may correspond to the coiling frequency of the jet. For example, the frequency of current fluctuations at 1 *kV* and 6 *kV* are around 1 *Hz* and 15 *Hz* respectively, as shown by the insets, while the corresponding coiling frequency are 1 *s^-1^* and 17.5 *s^-1^* respectively. The time interval of current measurement is 10^-3^ *s*. The scale bar is 2 *mm*. A lecithin jet with a flow rate of 50 *ml/h* is used for this plot.

1. **Comparison between electrical approach and geometric approach by varying the dispensing height *h***

The electric approach to manipulate coiling is advantageous to geometric control*,* especially in cases where the dispensing height, *h,* cannot be varied. More importantly, the range of the jet radius and coiling frequency enabled by the electrical approach is wider than that by the geometric one. With the geometric approach, as we increase *h*, the jet velocity increases and consequently the jet becomes thinner, leading to fast coiling^[1–6]^. However, when the circumference of the thin jet is smaller than the wavelength of the jet, the jet is unstable and tends to break up into smaller droplets due to Plateau–Rayleigh instability. The timescale that governs jet break-up is $t_{breakup}\sim\mu d/\gamma$(Ohnesorge number >1), where $\mu,d,\gamma$ are the viscosity, average jet diameter and surface tension of the fluid respectively ^[9]^. Thus the jet break-up time is determined by the fluid property and the jet radius. If the jet break-up time is smaller than the jet travelling time, $t_{travel}\sim h/v$, where *v* is the average jet velocity, the jet will break up into droplets instead of forming coils, when it approaches to the substrate (Fig. S3a). Assuming constant flow rate $Q\sim(d/2)^{2}\cdot v$, the jet will break up when $\mu d/\gamma<h/v$. Thus, the thinnest uncharged jet that can be reached is $r\sim\frac{2\mu Q}{\gamma h}$ (Fig. S3b).

Interestingly, for liquids with small conductivities (<10^-4^ *S/m*), an electric field applied along the axial direction can completely suppress the Plateau–Rayleigh instability ^[5-12]^. An otherwise unstable thin jet can now be stabilized by an axial electric field and thus breakup is prevented. As a result, a much thinner jet and thus a faster coiling frequency can be achieved in the presence of an electric field. For example, when a lecithin jet is dispensed from a nozzle of 0.92 *mm* at a height 25mm and a flow rate of 30 *ml/h*, the smallest radius reached before breakup is predicted to be around 0.4 *mm* in the absence of electric field (Fig. S3b). In the presence of an electric field, the jet radius can be below 0.1 *mm* without breakup under the same conditions (Fig. 1c). Therefore, the electrical force can facilitate formation of a thinner jet with a faster coiling frequency at a significantly shorter dispensing height, *h*.


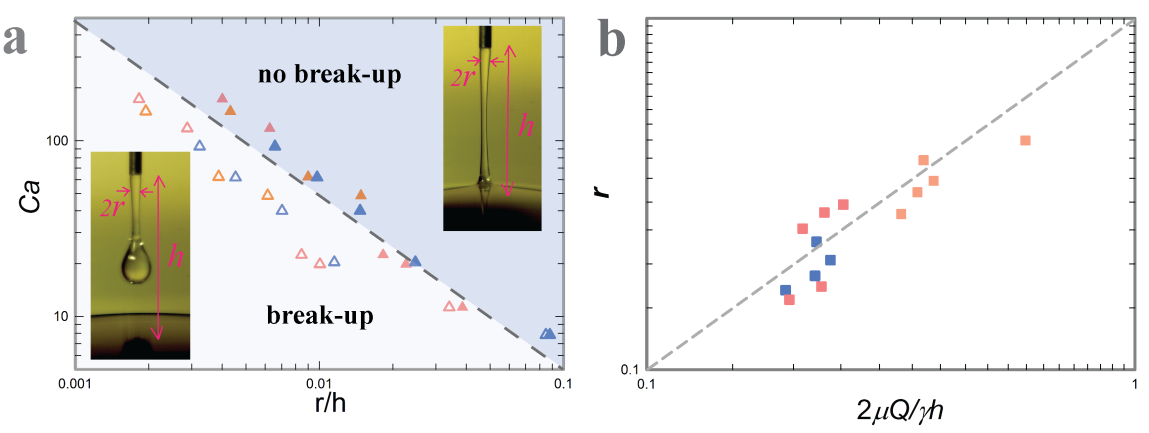


**Figure S3.** (a) Phase diagram for jet breakup summarized using two dimensionless numbers, capillary number of the viscous jet and the ratio of the jet radius, *r,* to the dispensing height, *h*. The solid and empty symbols denote breakup and no breakup respectively. b) The predicted and measured jet radius, *r*, before breakup. The predicted jet radius is calculated by $r\sim2\mu Q/\gamma h$, representing the gray dotted line. The cubic symbols denote the measured jet radius. The pink, orange and blue colors represent different fluids used for experiments, which are lecithin, silicone oil and polydimethylsiloxane respectively.

1. **Formation of rings pattern from the coiling of compound viscous jet**


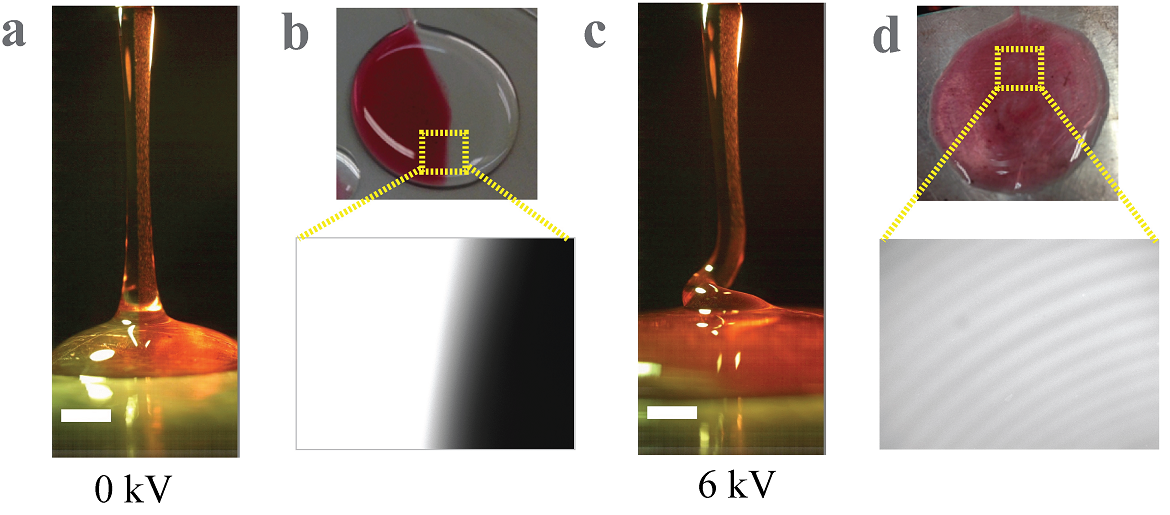


**Figure S4.** The two viscous silicone oils, with and without dye respectively, are injected into the nozzle, and flow in parallel until they fall onto the collection plate. With no electric field, a distinctive interfacial exists between the transparent and dyed regions, both for a) the falling jet and b) the resultant puddle attached on the collection plate. (c) With an appliedvoltage 6 *kV*, the viscous liquid jet is set to coil. The dispensing height is kept at 15 *mm*. (d) The deposition on the collection plate appears uniform macroscopically, but microscopically under fluorescence microscopy, a ring pattern is observed due to the different dye concentrations. All the images of the deposited puddle on the collection plate are taken immediately after the jet comes into contact with the substrate. In both cases, the flow rates of the two injected viscous liquids are kept constant at a flow rate of 40 *ml/h*. The same amount, 800 *µL*, of the silicone oils was collected for both samples.

1. **Heat flux *q* scales linearly with the reciprocal of diffusion distance**$\Delta L$

For diffusion-controlled reactions, the produced heat flux, *q,* during reaction scales linearly with the diffusion flux, *J*, of the reactants, $q\sim J$ . Moreover, by Fick’s first law, the diffusion flux, *J*, scales linearly with the reciprocal of the diffusion distance ,$\Delta L$.

The driving force for diffusion is the difference in concentration. Fick’s first law of diffusion relates the diffusive flux *J* to the concentration gradient , assuming steady state (Fig. S5).

(1)

where *D* is the diffusion coefficient, *J* is the diffusion flux, *x* represents position and Ф denotes concentration.

Applying Fick’s first law, the diffusion flux scales linearly with the reciprocal of diffusion distance, which is $\Delta L$ in our case, .

Therefore, the generated heat flux, *q*, scales linearly with the reciproal of the diffusion distance, $\Delta L$: $q\sim\Delta L^{-1}$.


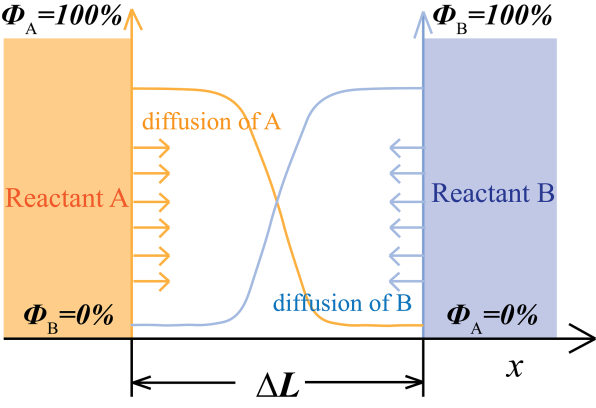


**Figure S5.** Schematic illustrating Fick’s first law of diffusion for two reactants

1. **Estimation of generated heat from a diffusion-controlled exothermic reaction using the internal heat equation and Newton's Law of Cooling**

In an inherently exothermic reaction as in our case, heat is generated within the puddle of epoxy. The heat generated from inside per unit time, per unit volume satisfy the following equation^[14]^:

$\frac{\partial T}{\partial t}=\alpha(\frac{\partial^{2}T}{\partial x^{2}}+\frac{\partial^{2}T}{\partial y^{2}}+\frac{\partial^{2}T}{\partial z^{2}})+\frac{1}{c_{p}\rho}\bar{q}$ (2)

where $\bar{q}$ is the generated heat per unit volume, per unit time (*J/m^3^s*); *T* represents temperature variations in space and time; *x,y,z* represents the Cartesian coordinates in space; $\alpha$, $c_{p}$,$\rho$ are the thermal diffusivity, specific heat capacity and density of the material respectively.

Since the temperature we measured is the average temperature of the deposited epoxy puddle, spatial variation is neglected, $\frac{\partial^{2}T}{\partial x^{2}}+\frac{\partial^{2}T}{\partial y^{2}}+\frac{\partial^{2}T}{\partial z^{2}}=0$. Thus we have

$\bar{q}=c_{p}\rho\frac{\partial T}{\partial t}$ (3)

By equation (3), we can calculate $\bar{q}$from the measured temperature change with time (Fig. 4a). However, while the temperature is monitored, the temperature difference between the epoxy puddle and the ambient temparture, *T_c_*, will lead to a certain amount of heat loss, $q_{loss}$ (*J/s*), to the ambient environment. This part of heat loss can be estimated using Newton's Law of Cooling (Fig. S6a)).

$q_{loss}=\lambda A(T-T_{c})$ (4)

where $\lambda$is the convective heat transfer coefficient, which depends on the physical properties of the materials as well as the physical situation where convection occurs; *A* is the surface area of the epoxy puddle. In our work, the convective heat transfer coefficient is experimentally measured.

In our experiments, after *t*=500 *s*, the exothermic reaction is completed, the main process is heat dissipation to the ambient environment, as indicated by the convergence of difference temperature curve in Fig. S6. Thus, we can assume that from *t*= 500 *s* onwards, the process is dominated by heat dissipation (Fig. S6a,b). From this part of curve, we can fit $\lambda$ as

$\lambda=\frac{c_{p}m(T_{2}-T_{1})}{(\int_{t_{1}}^{t_{2}} Tdt-T_{c}\Delta t)A}$ (5)

where *m* is the mass of the epoxy puddle.

Thus, the generated heat flux, *q* (*J/s*), of reaction should be the sum of heat generated per unit volume, $\bar{q}$(*J/m^3^s*), and the heat loss to the ambient environment, $q_{loss}$(*J/s*), $q=\overline{q}\cdot V+q_{loss}$, where *V* is the volume of the epoxy puddle.


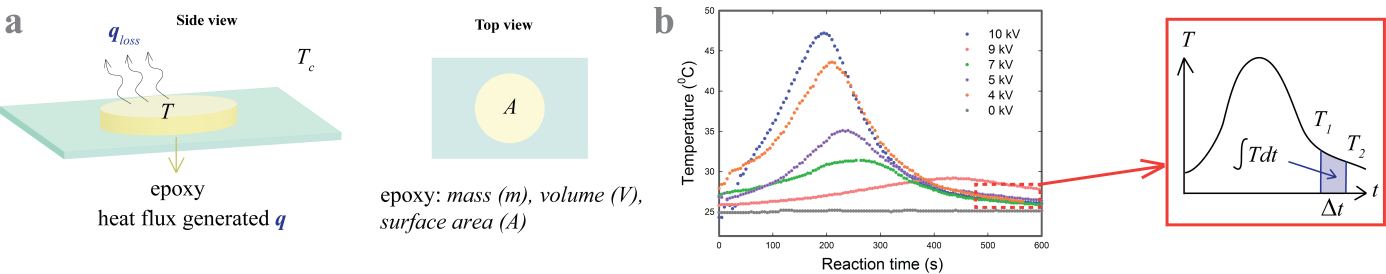


**Figure S6.** (a) Schematic showing the generated heat flux within the epoxy puddle and heat loss to the ambient environment; (b) A plot of the temperature as a function of reaction time. (inset) An sample plot illustrating the estimation of convective heat transfer coefficient, $\lambda$.

1. **Degree of mixing for epoxy**

The degree of mixing, *β*, for epoxy is defined as the change in enthalpy divided by the total change in enthalpy of the complete reaction, $\beta=\Delta H/\Delta H_{total}$^[15,16]^. The heat, *q,* generated during a chemical reaction at a constant pressure is equal to the change in the enthalpy of the system, $\Delta H$. Thus, the degree of mixing, *β*, for epoxy can be represented by the heat generated during reaction divided by the heat generated during a complete reaction.

1. **The comparison of the property of the mixture mixed with and without electric field**

**
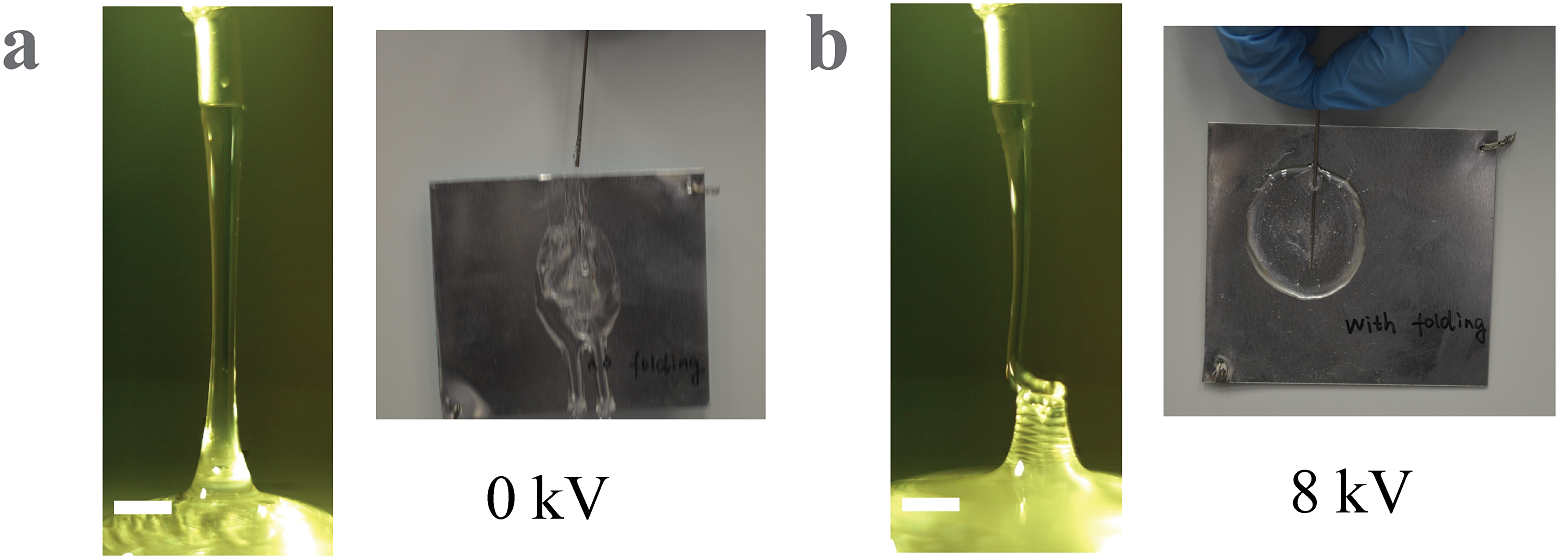
**

**Figure S7.** (a) Without electric field, the two reactive epoxy resins are poorly mixed, remain liquid-like, and thus cannot stick the metal rod to the plate (insert); (b) with an applied voltage 8 *kV*, the coiling of resins triggers mixing. The dispensing height is kept at 13.3 *mm*. The solidified resultant mixture enables adhesion of the metal rod to the plate (insert). In both cases, the flow rates of the two injected resins are kept constant at 25 *ml/h*. The scale bar is 2 *mm*.

1. **Fabrication of different dispensing nozzles**

The dispensing nozzle was fabricated by assembling glass capillaries or metal tubes depending on the applications. There were several ways to fabricate the nozzles that allow simultaneous injection of two or more liquids (Fig. S8).

1. We tapered the tip of a two-bore theta-shape capillary (World Precision Instruments), which has inner and outer diameters of 1.02 *mm* and 1.5 *mm* respectively, using a micropipette puller (Sutter P97). The tip of this tapered theta-shape capillary can be polished to a desired diameter using sand paper. The diameter of the nozzle tip ranged from 20 *µm* to 500 *µm*. Metal syringe needles, with an outer diameter of 0.92 *mm*, and polyethylene microtubings (Scientific Commodities Inc.), with an inner diameter of 0.84 *mm*, were used to inject the viscous liquids. A 5-minute epoxy was used to affix the assembled parts on a glass substrate. To introduce the electric field, we usually connected the metal syringe needles to the positive end of a high voltage power supply.

2. We inserted two identical metal tubes into a big metal tube, and glued them together using 5-minute epoxy. The inner and outer diameters of the nozzle tip were 1.4 *mm* and 1.7 *mm* respectively.

3. We also used a Y-shape connector (Cole-Parmer Instrument Co.) with an inner diameter of 1.58 *mm*. The two upper inlets of the Y-shaper connector were used to inject viscous liquids. The lower inlet of the Y-shape connector was connected to a metal tube to facilitate the introduction of the electric field. The inner and outer diameters of the nozzle tip were 0.8 *mm* and 1.4 *mm* respectively.

**
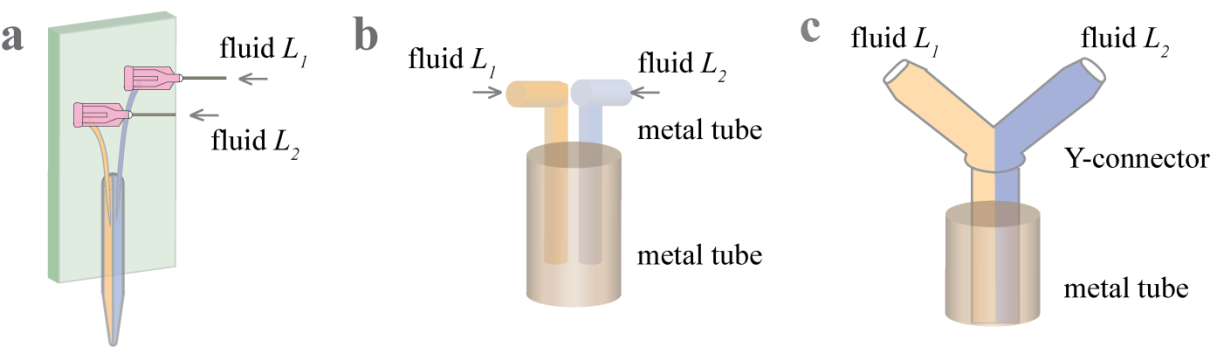
**

**Figure S8.** Schematic of dispensing nozzles based on assembling (a) glass capillaries, (b) metal tubes, and (c) Y-shape connector and metal tube

1. **Viscosity of liquid phases used**

The liquids employed in all experiments had a viscosity higher than 3 *Pa.s*; these included commercially available epoxy resins (Pattex, 5-minute), polydimethylsiloxane (Dow Corning), polyglycerolpolyricinoleate (PGPR, ILshinwells co., Ltd), silicone oil (Aladdin). The viscosities of the liquids used in our experiments were measured using a rheometer (Brookfield, R/S series) and were listed in Figure S9 and summerized in Table S10. All the viscous liquids we used in this manuscript are Newtonian, and thus non-Newtonian effects are not taken in account.


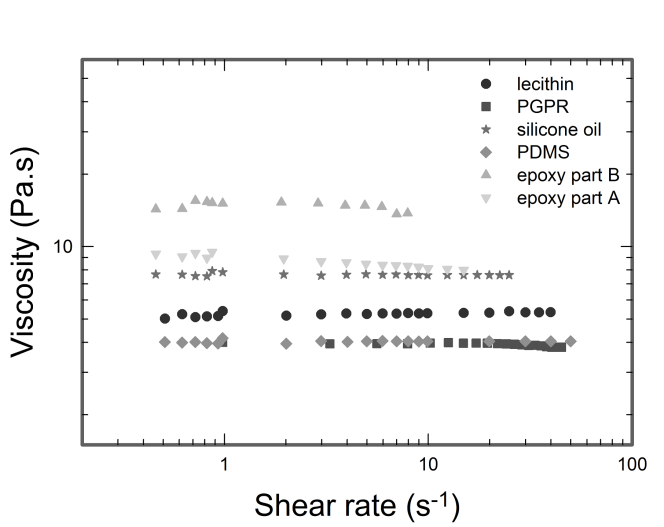


**Figure S9.** The measured viscosity of various viscous liquids at different shear rates. All experiments are conducted in the range of shear rates where the viscosity remains constant.

| Composition of liquid | Viscosity (*Pa.s*) |
| --- | --- |
| Lecithin from soy bean | 5.25 |
| Polydimethylsiloxane (PDMS) | 4.02 |
| Silicone oil | 7.64 |
| Epoxy resin part A | 8.42 |
| Epoxy resin part B | 14.11 |
| Polyglycerolpolyricinoleate (PGPR) | 3.92 |

**Table S10.** The viscosity of various viscous liquids

1. **A video demonstrating the coiling of a viscous jet**

A video recording a coiling viscous jet at an applied voltage of 3 kV and a dispensing height, 10 *mm*. The dispensed viscous jet is 18 wt% polycaprolactone (PCL) in chloroform at a flow rate of 2 *ml/h*.

1. **A video demonstrating the coiling of a compound viscous jet**

A video recording a compound jet, comprising two dyed silicone oils, red and green respectively, coils under different applied voltage from 0 kV to 7 kV. The dispensing height is kept constant, 13 *mm*. The flow rates of the two viscous liquids are kept constant at a flow rate of 40 *ml/h*.

**References:**

[1] L. Mahadevan, W. Ryu, A. Samuel, *Nature* 1998, 392, 140.

[2] N. M. Ribe, *Proc. R. Soc. A* 2004, 460, 3223.

[3] M. Habibi, Y. Rahmani, D. Bonn, N. M. Ribe, *Phys. Rev. Lett.* 2010, 104, 074301.

[4] N. M. Ribe, M. Habibi, D. Bonn, *Annu. Rev. Fluid Mech.* 2012, 44, 249.

[5] N. M. Ribe, H. E. Huppert, M. A. Hallworth, M. Habibi, D. Bonn, *J. Fluid Mech.* 2006, 555, 275.

[6] N. Ribe, M. Habibi, *Phys. Fluids* 2006, 18, 268.

[7] G. Taylor, *Proc. R. Soc. A* 1966, 291, 159.

[8] J. R. Melcher, W. J. Schwarz, *Phys. Fluids* 1968, 11, 2604.

[9] N. K. Nayyar, G. S. Murty, *Proc. Phys. Soc.* 2002, 75, 369.

[10] S. Sankaran, D. A. Saville, *Phys. Fluids* 1993, 5, 1081.

[11] D. A. Saville, *Phys. Fluids* 1970, 13, 2987.

[12] J. Eggers, E. Villermaux, *Reports Prog. Phys.* 2008, 71, 036601.

[13] E. Bormashenko, R. Pogreb, G. Whyman, A. Musin, *Colloids Surfaces A Physicochem. Eng. Asp.* 2009, 351, 78.

[14] J.P.Holman, *Heat and Mass Transfer*, McGraw-Hill Companies, 1996.

[15] J. M. Morancho, J. M. Salla, *Polymer* 1999, 40, 2821.

[16] B. Bilyeu, W. Brostow, K. P. Menard, *J. Mater. Educ*. 2000, 22, 107.
